# Management of open fractures of the tibial shaft in multiple trauma

**DOI:** 10.4103/0019-5413.43378

**Published:** 2008

**Authors:** Bołtuć Witold Stanisław, Golec Edward Bogusław

**Affiliations:** Department of Traumatic-Orthopaedic Surgery and Rehabilitation, ul. Szpitalna 1, 33-200 Dąbrowa Tarnowska, Poland; 1 Clinic of Traumatic Surgery and Orthopaedics, Department of Rehabilitation of V Military Clinical Hospital with Policlinic SP ZOZ, Kraków; Department of Physiotherapy, Institute of Physiotherapy, Faculty of Health Sciences, Jagiellonian University Medical College, Poland

**Keywords:** Intramedullary nailing, multiple trauma, open tibial shaft fracture

## Abstract

**Background:**

The work presents the assessment of the results of treatment of open tibial shaft fractures in polytrauma patients.

**Materials and Methods:**

The study group comprised 28 patients who underwent surgical treatment of open fractures of the tibial shaft with locked intramedullary nailing. The mean age of the patients was 43 years (range from 19 to 64 years). The criterion for including the patients in the study was concomitant multiple trauma. For the assessment of open tibial fractures, Gustilo classification was used. The most common concomitant multiple trauma included craniocerebral injuries, which were diagnosed in 12 patients. In 14 patients, the surgery was performed within 24 h after the injury. In 14 patients, the surgery was delayed and was performed 8–10 days after the trauma.

**Results:**

The assessment of the results at 12 months after the surgery included the following features: time span between the trauma and the surgery and complications in the form of osteomyelitis and delayed union. The efficacy of gait, muscular atrophy, edema of the operated limb and possible disturbances of its axis were also taken under consideration. In patients operated emergently within 24 h after the injury, infected nonunion was observed in three (10.8%) males. These patients had grade III open fractures of the tibial shaft according to Gustilo classification. No infectious complications were observed in patients who underwent a delayed operation.

**Conclusion:**

Evaluation of patients with open fractures of the tibial shaft in multiple trauma showed that delayed intramedullary nailing performed 8–10 days after the trauma, resulted in good outcome and avoided development of delayed union and infected nonunion. This approach gives time for stabilization of general condition of the patient and identification of pathogens from wound culture.

## INTRODUCTION

The problem of multiple trauma has been an everlasting concern of traumatologists. The algorithms of effective management of these difficult and prognostically uncertain injuries are still being searched for. The attempts of standardization of the terminology and classification of multiple injuries and their remote consequences are being undertaken. According to Gopal *et al*.,^1^,^2^ Robinson,^3^ and Court-Brown,^4^ open fractures require anatomical repositioning of the bone fragments and their fixation within 24 h after the injury with early covering of soft tissue loss. Is it really a proper management? The present work tries to evaluate it.

The aim of the present research is assessment of the results of management of open tibial shaft fractures in multiple trauma patients with locked intramedullary nailing, and finding answers to the following questions:

1. What is the relation between results of treatment of open tibial shaft fractures with intramedullary nailing, the time of operation, and the type of fracture according to Gustilo classification?
2. What are the most common complications of the management in observation, depending on the type of fracture according to the abovementioned classification?

## MATERIAL AND METHODS

The study group comprised 28 patients, of which eight (28.6%) were females and 20 (71.4%) males with diagnosed open tibial shaft fractures with concomitant multiple trauma, treated from year 1999 to 2006 at our institute. The mean age of patients was 43 years (range 19-64 years). The fractures were accompanied most frequently by craniocerebral injuries in 12 (43%) patients [Table 1]. For fracture assessment, Gustilo classification was adopted.^5^ The most common causes of open tibial shaft fractures and concomitant injuries were road accidents in 17 (60.7%) patients. Falls from height caused fractures in six (21.4%) patients. Accidents during farming work resulted in fractures in five (17.9%) patients

**Table 1:** Concomitant injuries depending on the type of fracture according to Gustilo classification

Concomitat injuries	Type of fracture according to Gustilo			Total
		
	II	IIIA	IIIB	
Craniocerebral injuries	7	3	2	12
Thoracic injuries	3	1	1	5
Fractures of the pelvis	1	1	–	2
Abdominal injuries	1	–	1	2
Other fractures of long bones	3	2	2	7
Total	15	7	6	28

The patients were divided into two groups, depending on the time of the operation. The first group included 14 (50%) patients [three (10.8%) females and 11 (39.2%) males] who were operated emergently, that is, within 24 h after the injury. The second group comprised of 14 (50%) patients [five (17.8%) females and nine (32.2%) males] who underwent delayed surgery, that is, after 8–10 days from the injury.

As per Gustilo classification, the first group included seven patients with type II fracture; four with type IIIA, and three with type IIIB, and in the second group, there were eight patients with type II fracture; three with type IIIA and three with type IIIB fracture.

Fractures of the tibial shaft were classified according to AO classification.^6^ There were 15 (53.6%) fractures of the type 42A, seven (25%) of the type 42B, and six (21.4%) of the type 42C. Open fractures were classified on the basis of the Gustilo classification. According to this classification, 15 (53.6%) fractures were of the type II, seven (25%) of the type IIIA, and six (21.4%) of the type IIIB [Table 2].

**Table 2:** Number of fractures according to AO classification in relation to type of fracture according to Gustilo classification

Type of fracture according to Gustilo	Type of fracture according to AO			Total
		
	42 A	42 B	42 C	
II	15	–	–	15
III A	–	4	3	7
III B	–	3	3	6
Total	15	7	6	28

Stabilization of the fracture with locked intramedullary nailing without intramedullary reaming was performed within 24 h after the injury in 14 (50%) patients, including three (10.8%) females and 11 (39.2%) males.

The wound after extensive irrigation and debridement of the tissues was covered with garamycin sponge and left open for checking after successive 24 and 48 h. Simultaneously, broad-spectrum antibiotics, anticoagulants, and vasodilators to improve circulation were administered. The subsequent revision of the wound assessed its clinical and bacteriological state. The wound was covered with artificial skin “codogard” until granulation developed and the base for a skin graft of medium thickness was prepared. Only in unconscious patients, the limb was immobilized with backslabs applied to prevent the development of contractures at the ankle.

Delayed osteosynthesis of the tibial shaft (8–10 days after the injury) was performed in 14 (50%) patients, of which five (17.8%) were females and nine (32.2%) males. Only the patients in stable general and local condition, with no clinical and laboratory features of inflammation and negative bacteriological cultures from the wound qualified for this type of surgery. In these patients, osteosynthesis of the tibial shaft fracture with locked intramedullary nailing was carried out without reaming of the medullary cavity. Dynamization of the union was performed 6-12 weeks after the operation, depending on the type of fracture and progress of union. Loading of the operated limb was begun approximately 4-6 weeks after the surgery, also depending on the type of fracture and rate of union.

In our study, radiological and clinical results were assessed. The radiological analysis included features of the fracture healing, assuming the following:

delayed union as the absence of fracture healing after 12-16 weeks from osteosynthesis; nonunion as the absence of union after 24-28 weeks from osteosynthesis.

Moreover, in the radiological examination features of possible inflammatory processes in the form of osteomyelitis was also taken under consideration.

The axis of the operated limb was assessed on the radiographs performed in anteroposterior and lateral projections. For this purpose, a line at a right angle to the middle of the plateau of the tibia and its articular surface and to the middle of the ankle was drawn. In the lateral projection, a line parallel to the anterior border of the tibia was drawn.

Clinical assessment included quality of gait, muscular atrophy, edema of the operated limb, and the range of knee and ankle mobility.

## RESULTS

The obtained results were analyzed in relation to the type of fracture according to Gustilo and Anderson classification 12 months after the surgery [Table 3].

**Table 3:** Infectious complications and delayed union as regards type of fracture according to Gustilo and time of surgery

Type of fracture according to Gustilo	Infected pseudarthrosis		Delayed union		Total	
			
	Group I %	Group II %	Group I %	Group II %	N	%
II	–ca	–	2 (7.2)	1 (3.6)	3	10.8
III A	1 (3.6)	–	1 (3.6)	1 (3.6)	3	10.8
III B	2 (7.2)	–	2 (7.2)	1 (3.6)	5	18
Total	3 (10.8)	–	5 (18)	3 (10.8)	11	39.6

### Gustilo type II

In this type of fractures, no significant differences as regards the time of operation were found, and no infections were noted in all study groups. Union was observed 16-28 weeks after the surgery (mean 20 weeks).

Delayed union occurred in three (10.8%) patients, of which two were in the group I, and one in the group II. In two patients, dynamization of the fixation effectively activated osteogenesis leading to union, and in one patient, exchange of the fixation was necessary after intramedullary reaming. No axial disturbances were observed in the operated tibia. Furthermore, no edemas and no marked muscular atrophy in the operated limb were noted; range of the motion of the normal knee and ankle was good, which ensured efficient and painless gait.

### Gustilo type IIIA

The mean time of union was 28 weeks. Delayed union was noted in two patients, each of them belonging to different groups. In the patient belonging to the second group, stimulation of osteogenesis was achieved after dynamization of the fixation, that is, after 14 weeks. The patient who underwent the operation within 24 h after the injury, stimulation of osteogenesis required another operation with the exchange of an intramedullary nail and reaming of the medullary cavity. In this case, union occurred 28 weeks after the second surgery.

In one patient from the first group, infected nonunion was noted in the 16th week after the operation. In this case, debridement of the infection site, seqestrectomy of nonvital tissue, and local intravenous antibiotic therapy was instituted without removal of the nail. After union 40 weeks later, the implant was removed, and the medullary cavity was reamed and irrigated with saline and gentamicin, which resulted in the remission of infection.

In two patients from the first group, considerable muscular atrophy and restriction of the flexion of the knee to 100° in the operated limb was noted. In one patient from this group who developed an inflammation, varus bending of the long tibial axis of 20° occurred.

### Gustilo type IIIB

The mean age of the patients with diagnosed open tibial bone fracture type IIIB was 42 years. Three (10.8%) of them belonged to the first group and three (10.8%) to the second group. In this type of fractures, no bone loss exceeding 25% of the circumference of the tibial shaft was noted. Indirect fragments, despite their poor blood supply, were not removed during the primary revision of the wound because of highly energetic mechanism of the trauma. All the fractures were fixed statically. Post-traumatic wounds after debridement and irrigation were left open. In three patients, 14 days after the operation, skin graft of medium thickness was necessary. In one patient, the secondary suturing of the post-traumatic wound was performed; in two patients, the wound healing was achieved through granulation.

In three patients, delayed union was noted of which two patients belonged to the first group and one to the second group. In two patients belonging to the first group, infected nonunion was observed in the 14th week of treatment. The etiological factor was *Staphylococcus aureus* and *Staphylococcus epidermidis*. Because of this, the exchange of the nails was carried out together with reaming and irrigation of the medullary cavity with saline and gentamicin, accompanied by local intravenous antibiotic therapy according to Court Brown.^7^ Union was achieved in the 52nd week of treatment. In the patients belonging to the second group, no infected nonunion was noted and union occurred approximately after 42 weeks.

In two patients, excessive external rotation of the tibia, resulting from improper fixation of bone fragments was found. In one patient belonging to the first group, varus twisting of the long tibial axis of 15° occurred, resulting from the use of the intramedullary nail of too small diameter in relation to the size of the medullary cavity and break of the locking screws.

## DISCUSSION

The advantages of stable osteosynthesis, is well known and accepted in multiple trauma patients. It ensures not only the treatment of the injury, but it is also beneficial for nursing of the patients, especially the unconscious ones and their physiotherapy.^8^–^11^ The role of factors facilitating the process of bone healing, as well as, those disturbing the patient's general condition and, therefore, influencing the quality and duration of the patient's recovery should be taken into consideration. Post-traumatic shock, systemic disturbances of the hemodynamic, metabolic, humoral and immunological origin, as well as local impairment of the tissue circulation promote infectious complications and their remote consequences.^8^,^9^,^12^ Multitrauma patients are usually hospitalized in the intensive care units, where they are exposed to multiresistant bacterial flora.^13^ Moreover, their general condition depends on the past and current systemic diseases, past operations, and the type of medicines taken. All these factors undoubtedly influence the course of the patients' treatment, especially when they require emergent surgery.

Adamczak,^14^ among others, is of the opinion that impairment of the blood flow in bone fragments, which is directly related to the mechanism of fracture, has a fundamental role in the development of post-traumatic osteomyelitis.

Fractures caused by a direct mechanism are usually multilevel, multifragmentary, and in many cases, the open fractures of the II and III grade, leading to various types of complications, including infections.^7^,^13^–^15^ Therefore, appropriate primary treatment of the soft tissues and stabilization of the bone fragments can prevent complications.^3^,^9^ Nowadays, intramedullary stabilization is a commonly accepted standard surgical management of both closed and open fractures of the type I, II, IIIA, and IIIB according to Gustilo–Anderson classification.^1^,^2^,^10^,^16^–^21^ Locked intramedullary nails are most commonly used because of small traumatization of the tissues, good stabilization of the bone fragments and possibility of their fixation without impairment of blood supply. According to Robinson,^3^ additionally, this type of management allows easier and more effective secondary procedures on the soft tissues, decreases the percentage of delayed union, and significantly reduces the risk of infections. According to Court Brown *et al*.,^4^ treatment of open tibial shaft fractures with locked intramedullary stabilization resulted in considerable improvement of infectious complications and delayed union. The necessity of the use of bone grafts in type IIIA fractures decreased to zero, and in type IIIB fractures decreased from 71% to 15% when compared with the results of treatment of the same type of fractures with the external stabilization. This was further confirmed by our observations of this type of treatment with no requirement of free bone grafts.

There are some discrepancies in the opinion of many authors^16^,^22^,^23^ concerning the time of the operation, especially in case of open fractures. In patients with multiple trauma, emergent stabilization of the fracture is beneficial for the patient's nursing and rehabilitation procedures.^18^,^22^ In our study, in some patients with open tibial shaft fractures, the surgery was delayed until healing of the wound and stabilization of the patient's general condition was achieved.

Observation of the results of the delayed surgery showed it gives definitely better radiological and clinical results than in the case of the emergent surgery. Undoubtedly, control of the injuries to the internal organs, causing internal hemorrhage or intracerebral bleeding followed by increased intracerebral blood pressure is more urgent than fixation of the fracture. Furthermore, stabilization of the patient's general condition, ensuring normal functioning of the vital organs is an indispensable element of life saving procedures, which allow effective delayed operations.

The delayed operation enables surgical and bacteriological inspection of the wound, its secondary revision, as well as supervision of the quality of the adjacent soft tissues, which creates optimal conditions for safe fixation of the bone fragments.^10^ Therefore, in our opinion, the decision on emergent fixation of open tibial shaft fracture should be made on the basis of the patient's general condition with properly functioning vital organs. It favors, in our opinion, initiation of soft tissue healing, avoids impairment of blood supply to the indirect bone fragments, the removal of which during the primary treatment of the wound is not always recommended.

The correct primary static stabilization of the fracture with intramedullary nailing enables further reconstruction of the indirect fragments, which initially have insufficient blood supply. This type of management did not increase the number of infections and union disturbances. The bone sequestration was noted only in one patient. However, duration of union was prolonged in open type IIIB fractures according to Gustilo–Anderson classification, as well as in fractures type 42C according to AO classification. Similar findings were presented by Keating and Court-Brown.^4^,^19^

Infectious complications in the patients who underwent emergent surgery developed several months after the surgery with proper primary healing of the soft tissues and bones. In our view, prophylactically instituted both general and local antibiotic therapy could cause chronic inflammatory process without the acute phase. Fibak^18^ in 1994 pointed to an increased number of infected nonunions after intramedullary stabilization of open fractures treated within 24 h after the injury. This led to delay in operation in cases of open fractures from 10 to 15 days. Gopal *et al*.^1^ state that multifragmentary compound fractures of the tibial type IIIB and IIIC according to Gustilo–Anderson classification should be treated surgically in the firm and aggressive way. It should be done by teams comprising orthopedists and plastic surgeons with the use of an intramedullary nail or external fixator and immediate covering of the skin loss with pedicled free or rotational muscle flaps. In the opinion of these authors, the use of external stabilization makes plastic surgery difficult and increases the rate of delayed union or nonunion. For this reason, as they state, fractures of this type should be treated only in specialist centers.

Similar opinion is presented by Naique,^24^ who evaluated the patients taken directly to specialist trauma centers and local hospitals and found that the majority of union complications, failure in the use of pedicle muscle flaps, and infectious complications occurred in patients treated in local hospitals.

Robinson *et al*.,^3^ say that only adequate blood supply of the bone fragments ensured by covering of the lost soft tissues can prevent the development of infection. According to these authors, the type of fracture is as important as the accompanying bone loss, which prolongs osteogenesis, and in the majority of patients, requires additional surgical procedures, usually bone grafting. In our study, there was no bone loss requiring grafts, and the remaining indirect bone fragments healed well.

Malik *et al*.,^25^ while examining the factors influencing the occurrence of bone infections and complications of union in various types of fractures, found that open fractures are significantly more prone to infections and union disturbances than the closed ones. They also point to the effect of invasiveness of particular types of fixation and stabilization on the frequency of complications.

## CONCLUSIONS

Delayed fixation of the open fractures of the tibial shaft in multitrauma patients gave significantly better radiological and clinical results when compared with emergent fixation.

The most common complications of the surgical treatment of open fractures of the tibial shaft in multitrauma patients with locked intramedullary nail were union disturbances and infections.
